# Tibial tunnel placement in posterior cruciate ligament reconstruction: a systematic review

**DOI:** 10.1007/s00167-013-2563-3

**Published:** 2013-06-16

**Authors:** J.-D. Nicodeme, C. Löcherbach, B. M. Jolles

**Affiliations:** Department of Orthopaedic Surgery and Traumatology, Site Hôpital Orthopédique, Centre Hospitalier Universitaire Vaudois and University of Lausanne, Avenue Pierre Decker 4, Lausanne, 1011 Switzerland

**Keywords:** Posterior cruciate ligament, Reconstruction, Retrospinal, Systematic review, Tibial tunnel placement

## Abstract

**Purpose:**

Reconstruction of the posterior cruciate ligament (PCL) yields less satisfying results than anterior cruciate ligament reconstruction with respect to laxity control. Accurate tibial tunnel placement is crucial for successful PCL reconstruction using arthroscopic tibial tunnel techniques. A discrepancy between anatomical studies of the tibial PCL insertion site and surgical recommendations for tibial tunnel placement remains. The objective of this study was to identify the optimal placement of the tibial tunnel in PCL reconstruction based on clinical studies.

**Methods:**

In a systematic review of the literature, MEDLINE, EMBASE, Cochrane Review, and Cochrane Central Register of Controlled Trials were screened for articles about PCL reconstruction from January 1990 to September 2011. Clinical trials comparing at least two PCL reconstruction techniques were extracted and independently analysed by each author. Only studies comparing different tibial tunnel placements in the retrospinal area were included.

**Results:**

This systematic review found no comparative clinical trial for tibial tunnel placement in PCL reconstruction. Several anatomical, radiological, and biomechanical studies have described the tibial insertion sites of the native PCL and have led to recommendations for placement of the tibial tunnel outlet in the retrospinal area. However, surgical recommendations and the results of morphological studies are often contradictory.

**Conclusions:**

Reliable anatomical landmarks for tunnel placement are lacking. Future randomized controlled trials could compare precisely defined tibial tunnel placements in PCL reconstruction, which would require an established mapping of the retrospinal area of the tibial plateau with defined anatomical and radiological landmarks.

**Level of evidence:**

III.

## Introduction

Posterior cruciate ligament (PCL) surgery has evolved significantly in recent years. Based on advanced anatomy and biomechanics, new surgical techniques have been developed to restore native knee kinematics and to control posterior laxity. Single-bundle or double-bundle PCL reconstruction can be performed using a tibial tunnel or inlay technique [40].

The inlay technique was popularized by Berg [3] in 1995 and requires a posterior knee approach. It has the advantage of direct visualization of the insertion of the PCL for an anatomical placement of the graft and avoids the so-called killer turn of the tendon transplant.

The tibial tunnel technique requires the placement of a tunnel into the retrospinal area. This exclusively arthroscopic surgery avoids posterior capsulotomy, which may induce additional laxity [31]. The entire procedure can be performed on a patient in the supine, flexed-knee position.

Accurate tibial tunnel placement is crucial for successful PCL reconstruction using arthroscopic tibial tunnel techniques. A discrepancy between anatomical studies of the tibial PCL insertion site and surgical recommendations for tibial tunnel placement remains. The results of PCL reconstruction remain inconsistent despite a large choice of operative techniques [5, 15, 23]. There is consensus that, for single-bundle reconstruction, the femoral tunnel should be placed at the anterolateral or at the central part of the footprint, rather than in the posteromedial aspect of the footprint to optimize laxity control (central part) and graft constraint (anterolateral part) [26]. However, recommendations for placement of the tibial tunnel are contradictory. The purpose of this study was to elucidate the optimal placement of the tibial tunnel in PCL reconstruction based on a systematic review of clinical studies, in order to optimize laxity control and improve outcomes.

The objective of this study was to identify the optimal placement of the tibial tunnel in PCL reconstruction based on clinical studies.

## Materials and methods

### Literature search

A search of the Cochrane Bone, Joint and Muscle Trauma Group database of systematic reviews (1990–2011), the Cochrane Central Register of Controlled Trials (September 2011), MEDLINE via PubMed (1990 to September 2011), and EMBASE (1990 to September 2011) using the key words “posterior,” “cruciate,” “ligament,” and “adult” was conducted. It included all clinical trials comparing two different tibial tunnel placements in the retrospinal area for PCL reconstruction using the tibial tunnel technique. The search was limited to studies in adult patients with PCL injury requiring a graft reconstruction. Particular attention was paid to the description of the tunnel placement in the retrospinal area and the anatomical landmarks used for placement. The search was restricted to English, French, Spanish, German, and Italian language publications. The original search strategy is shown in “Appendix”.

The three authors independently reviewed the abstracts of all publications identified by the literature search strategy. Studies that did not compare at least two different techniques of PCL reconstruction were excluded from review. All three authors reviewed the remaining publications individually. Consensus was reached through discussion of any disagreements.

### Statistical analysis

Counts of retrieved articles were tabulated. Reasons for exclusion were documented.

## Results

**Fig. 1:**
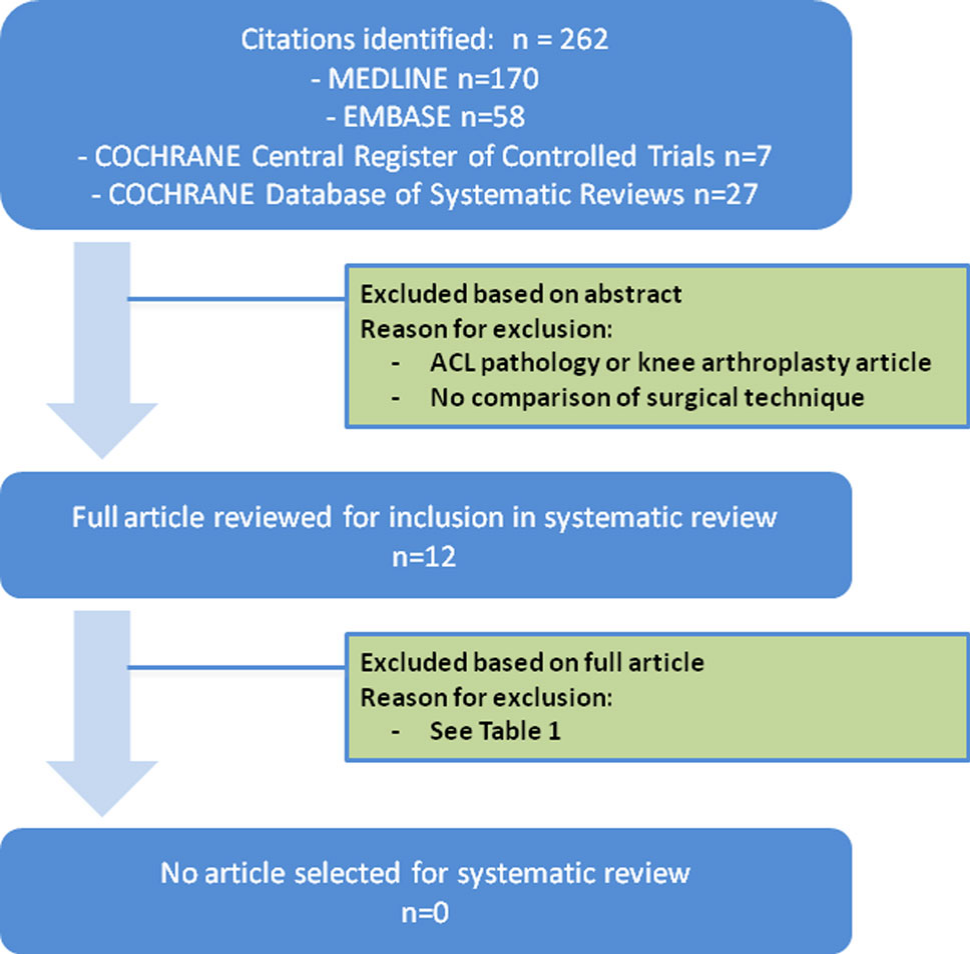
Search strategy and results for systematic review of the literature

The initial search strategy identified 262 publications (Fig. 1). Twelve clinical trials compared at least two different surgical techniques for PCL reconstruction (Table 1); none compared graft placements in the retrospinal area using a tibial tunnel technique. Ten anatomical studies, two radiological studies, and three biomechanical studies evaluating the tibial insertion site of the PCL were identified.

**Table 1:** Clinical trials that compared two or more surgical techniques for PCL reconstruction and reasons for exclusion from final analysis

	Techniques compared	Study design	Number of patients	Minimum follow-up	Tibial tunnel placement	Reasons for exclusion
Ahn et al. [1]	Hamstring tendon autograft versus Achilles tendon allograft	Retrospective case–control	36	2 Years	No description	No variation of tibial tunnel position
Chen et al. [5]	Quadriceps versus quadruple hamstring PCL reconstruction	Retrospective case series	49	2 Years	Distal and lateral on footprint	No variation of tibial tunnel position
Freeman et al. [11]	With or without posterolateral corner reconstruction	Retrospective case series	17	14 Months	No description	No variation of tibial tunnel position
Hatayama et al. [16]	Single- versus double-bundle PCL reconstruction	Retrospective case series	20	2 Years	No description	No variation of tibial tunnel position
Jung et al. [18]	Fibular head or tibial tunnel for posterolateral corner reconstruction	Retrospective case series	39	2 Years	No description	No variation of tibial tunnel position
Kim et al. [20]	Tibial tunnel single versus inlay single versus inlay double	Retrospective case series	29	2 Years	No description	No variation of tibial tunnel position
Kim et al. [21]	1 versus 2 incision PCL reconstruction	Retrospective case series	55	2 Years	1.5 cm below the articular margin	No variation of tibial tunnel position
Li et al. [22]	Hamstring graft versus LARS artificial ligament	Retrospective case series	36	2 Years	Distal and lateral on footprint, 8–10 mm from articular joint	No variation of tibial tunnel position
MacGillivray et al. [25]	Tibial inlay versus tibial tunnel technique	Retrospective case series	20	2 Years	No description	No variation of tibial tunnel position
Wang et al. [41]	Autograft versus allograft PCL reconstruction	Prospective randomized study	55	2 Years	1 cm below the articular surface of the medial plateau	No variation of tibial tunnel position
Wang et al. [42]	Single- versus double-bundle PCL reconstruction	Prospective randomized study	35	2 Years	1 cm below the articular surface of the medial plateau	No variation of tibial tunnel position
Wong et al. [43]	Anteromedial versus anterolateral transtibial approach	Prospective randomized study	55	3 Years	1 cm below the articular surface of the medial plateau	No variation of tibial tunnel position

*LARS* ligament augmentation and reconstruction system

Ten anatomical studies utilized various anatomical landmarks to describe the tibial insertion site of the PCL or its two bundles (Table 2). Girgis et al. [13] located the PCL insertion site in the depression behind the interarticular upper surface of the tibia, with a few millimetres extension onto the adjoining posterior surface of the tibia. Takahashi et al. [38] documented the tibial insertion site of both PCL bundles on 33 tibiae, using the anterior margin of the tibia, the medial border of the tibial plateau, and the vertical distance from the tibial plane as reference points. Using the same anatomical reference points, Tajima et al. [37] reported that the individual tibial insertion sites of both PCL bundles were in different planes on the posterior intercondylar fossa. Greiner et al. [14] determined the PCL insertion site using computed tomography scans and an additional anatomical reference, the posterior edge of the retrospinal surface.

**Table 2:** Placement of the PCL tibial insertion according to anatomical studies

Study	Study methodology	Number of knees	Posterior cruciate ligament		
			PCL centre	Antero-lateral bundle centre	Posterio-medial bundle centre
Cosgarea et al. [8]	Review study	n/a	10–15 mm under the articular surface of the tibia		
Edwards et al. [9]	Cadaveric dissection	39		Posterior horn of the medial meniscus is the anterior edge of AL bundle 37 mm from the medial edge of the plateau	7 mm under the articular surface of the tibia 38 mm from the medial edge of the plateau
Girgis et al. [13]	Dissection of cadaveric and fresh knees	44	On the retrospinal surface Extended for a few millimetres onto the adjoining posterior surface of the tibia		
Greiner et al. [14]	CT scans of dissected cadaveric knees	10	1.6 mm inferior to the articular surface of the plateau 46.1 mm from the anterior margin of the plateau 36.6 mm from the medial edge of the plateau 49 % of the width of the plateau		
Moorman et al. [28]	Sectioning and radiographic analysis of cadaveric knees	14	7 mm in front of the tibial posterior cortex		
Ramos et al. [33]	Cadaveric dissection	30	15 mm under the articular surface of the tibia 10.3 mm in front of the posterior capsule		
Sheps et al. [35]	Cadaveric dissection	10	Distal to cartilage tidemark and posterior horns of menisci Proximal to palpable cortical ridge in PCL fossa		
Tajima et al. [37]	Cadaveric dissection	21		1.5 mm under the articular surface of the tibia 34.3 mm from the medial edge of the plateau 41.3 mm from the anterior margin of the plateau 47 % of the width of the plateau	6 mm under the articular surface of the tibia 31.8 mm from the medial edge of the plateau 47.1 mm from the anterior margin of the plateau 44 % of the width of the plateau
Takahashi et al. [38]	Cadaveric dissection	33		The same level as the articular surface of the tibia 48.2 mm from the medial edge of the plateau. 51 % of the width of the plateau	4.6 mm distal to the articular surface of the tibia 47.4 mm from the medial edge of the plateau 50 % of the width of the plateau
Van Dommelen et al. [39]	Review study	n/a	10 mm distal to the articular surface of the tibia		

In a radiological study, Racanelli and Drez [32] reproducibly identified PCL tibial attachment superior to and onto the posterior tibial ridge, and 2–3 mm lateral to the centre of the lateral tibial tubercle, with an error margin of 2.5 mm. Similarly, Lorenz et al. [24] reported that the geometric centre of the tibial insertion was located at 51 ± 2 % of the total mediolateral width of the tibial plateau. In the sagittal plane, this point was 13 ± 2 % below the medial plateau tangent, using the total sagittal diameter of the tibial plateau as a reference.

Three biomechanical studies compared different graft placements in the PCL fovea and their impact on anteroposterior laxity control [4, 12, 27]. Galloway et al. [12] tested five tibial graft placements in the PCL fovea. The femoral insertion was placed at the isometric point, and the tibial insertion was moved either in the sagittal or frontal plane. There was no significant difference in anteroposterior laxity between the more anterior and posterior tunnel placement. A significant difference in laxity was found between medial and lateral placements from 30° to 60° of knee flexion: lateral displacement yielded better laxity control, but increased stress on the joint between 0° and 50° of flexion. Bomberg et al. [4] corroborated that tibial attachment variation in the sagittal plane had minor effects on graft isometry. Markolf et al. [27] placed the femoral tunnel 5 mm distal to the geometric centre of the femoral PCL insertion, to simulate anterolateral bundle reconstruction. The tibial tunnel was positioned 5 mm medial or lateral to the geometric centre of the tibial insertion. Errors in mediolateral tunnel position did not significantly influence laxity control between 5° and 120° of knee flexion. However, medial displacement of the tunnel led to increased graft forces beyond 65° of flexion.

## Discussion

The most important finding of the present study is the lack of clinical research-based evidence for an optimal tibial tunnel placement in PCL reconstruction using the tibial tunnel technique. No clinical trial matched the inclusion criteria for the study. Several recommendations based on anatomical, radiological, or biomechanical investigations were identified in the literature [2, 4, 6–10, 12–14, 24, 25, 27, 28, 32–39], but they are sometimes contradictory and do not match the surgical recommendations of medical textbooks.

Cadaveric studies utilized various anatomical landmarks to describe the PCL insertion site. This probably reflects the difficulty in finding consistent and reliable landmarks. Many techniques used only one reference value, although at least two coordinates are necessary to define a point geographically, and more are needed for an accurate three-dimensional mapping as proposed by Tajima et al. [37], Takahashi et al. [38] and Greiner et al. [14]. These studies provided detailed descriptions of the tibial PCL insertion, but the anatomical landmarks proposed are not always suitable for arthroscopic surgery with the patient in supine position.

Radiological studies also attempted to identify landmarks for definition of the PCL tibial insertion site [24, 32]. However, they did not rely on identical reference points and did not distinguish between the anterolateral and posteromedial bundles. Two more recent radiological studies have distinguished between the two PCL bundles. Osti et al. [30] correlated radiography and descriptive anatomy and observed that the cross-sectional areas and femoral and tibial insertions for the anterolateral and posteromedial bundles were similar to, but smaller in area than those observed anatomically by Takahashi et al. [38], and the intercondylar depth of the two bundles was smaller than that observed radiologically by Lorenz et al. [24], with the insertion areas deeper into the intercondylar wall. Johannsen et al. [17] characterized the anterolateral and posteromedial bundles of the PCL radiologically and recommended that a single tibial tunnel should be located between 1 and 2 mm distal to the joint line on the anteroposterior view. It is not yet known whether this location is consistently reproducible during arthroscopic PCL reconstruction surgery and leads to effective maintenance of joint stability.

The biomechanical studies reviewed [4, 12, 27] did not provide sufficient data to identify the optimal placement of the PCL tibial insertion for all degrees of knee flexion.

Several medical textbooks were also reviewed and demonstrated considerable variation in recommendations for tibial tunnel placement. Noyes et al. [29] and Strobel [36] placed the tibial guide at 12–20 mm distal to the joint line. Fanelli [10] suggested placement on the distal part of the PCL fovea to avoid the “killer turn” for the tendinous graft. Christel et al. [6] recommended placement in the distal third of the retrospinal area. Sekiya et al. [34] recommended that the transtibial guide pin should be placed 1 cm below the joint line. Kantaras and Johnson [19] suggested drilling the tibial tunnel distal and lateral to the medial meniscal root. Finally, Badet and Siegrist [2] positioned the tip of the guide 1.5 cm below the articular surface. However, none of these authors could rely on clinical evidence to inform their chosen placement of the tibial tunnel placement.

There is still a mismatch between surgical recommendations for tibial tunnel placement and biomechanical, radiological and anatomical data. This may be due to certain technical issues, such as prevention of the “killer turn” for the tendinous graft. Biomechanical studies show that anterior and posterior tibial tunnel position is less important than medial and lateral placement for laxity control, but they do not reflect behaviour of the graft in vivo. Different tunnel placements may change the length of the free intra-articular graft and the stiffness of the reconstruction and thus alter laxity control. Radiological landmarks may be helpful for tunnel placement, but have limited accuracy due to imaging quality within the surgical setting and use of simple two-dimensional images.

There was no significant difference in anteroposterior laxity between the more anterior and posterior tunnel placement. However, a significant difference in laxity was found between medial and lateral placements from 30° to 60° of knee flexion; lateral displacement yielded a better laxity control, but increased stress on the joint between 0° and 50° of flexion.

## Conclusions

This systematic review did not identify an optimal tibial tunnel placement for arthroscopic PCL reconstruction using a tibial tunnel technique. In the absence of other clinical evidence, tunnel placement for PCL reconstruction should be anatomical as for ACL reconstruction. A detailed cartography of the PCL fovea is necessary to establish consistent, reproducible anatomical landmarks for surgery. Randomized clinical trials comparing at least two defined positions of the tibial tunnel graft on the retrospinal area during PCL reconstruction are needed, to determine whether the positions can be consistently achieved and result in effective, reliable maintenance of joint stability, and to evaluate complication rates.

## Appendix

### Original search strategy for MEDLINE (OVID)

#### Terms

- (“posterior cruciate ligament”[MeSH Terms] OR (“posterior”[All Fields] AND “cruciate”[All Fields] AND “ligament”[All Fields]) OR “posterior cruciate ligament”[All Fields]) OR PCL[All Fields] AND (Clinical Trial[ptyp] AND (English[lang] OR French[lang] OR German[lang] OR Italian[lang] OR Spanish[lang]) AND “adult”[MeSH Terms] AND (“1990/01/01”[PDAT]: “2011/10/01”[PDAT]))

#### Limits

- Clinical trials
- All adult: +19 years
- English, German, Italian, French, Spanish
- January 1990–September 2011

#### Number

- 170

### Cochrane database of systematic reviews

#### Terms

- Posterior cruciate ligament (all field)

#### Number

- 27

### Cochrane central register of controlled trials

#### Terms

- Posterior cruciate ligament (all field)

#### Number

- 7

### Original search strategy for EMBASE

#### Terms

- Posterior AND cruciate AND ‘ligament’/exp AND [controlled clinical trial]/lim AND ([english]/lim OR [french]/lim OR [german]/lim OR [italian]/lim OR [spanish]/lim) AND ([adult]/lim OR [aged]/lim) AND [1990–2011]/py

#### Limits

- Controlled clinical trials
- January 1990–September 2011
- Age: 18–64, 65, and more
- Language: English, German, Italian, French, Spanish

#### Number

- 58
